# Trees in Towns

**Published:** 1888-03-24

**Authors:** 


### TREES IN TOWNS.

TREES in streets temper the heat and serve as a protection from dust, and the evaporation from their leaves tends to keep the surrounding air cool and moist; while the perpetual vibration of their foliage and swaying of their branches, whilst admitting a sufficient amount of light, serve to protect the eyes from the noon-day glare. Moreover, the roots of trees draw up stagnant waters, and absorb the organic matters contained in the filth from which the streets of a town are never free; and which, after infiltrating the ground, are a frequent cause of fevers and infection. Trees, in fact, have the same effect on the subsoil of towns as fields have on the contents of their sewers—they act as disinfectants. The planting of trees in our streets and squares is to be encouraged, not only because of their beauty, but becau e of their hygienic value. The practice which prevails, however, in many Continental towns of cutting the trees down to a uniform size is to be deprecated; as the branches of trees so treated become so impacted as to be impervious to light, and the shade they give under such circumstances, although deep, is too circumscribed to be of much use.

